# Binder-free graphene oxide doughs

**DOI:** 10.1038/s41467-019-08389-6

**Published:** 2019-01-24

**Authors:** Che-Ning Yeh, Haiyue Huang, Alane Tarianna O. Lim, Ren-Huai Jhang, Chun-Hu Chen, Jiaxing Huang

**Affiliations:** 1 Department of Materials Science and Engineering, Northwestern University, Evanston, IL 60208 USA; 2 Department of Chemistry, National Sun Yat-sen University, Kaohsiung, 80424 Taiwan

## Abstract

Graphene oxide (GO) sheets have been used to construct various bulk forms of GO and graphene-based materials through solution-based processing techniques. Here, we report a highly cohesive dough state of GO with tens of weight percent loading in water without binder-like additives. The dough state can be diluted to obtain gels or dispersions, and dried to yield hard solids. It can be kneaded without leaving stains, readily reshaped, connected, and further processed to make bulk GO and graphene materials of arbitrary form factors and tunable microstructures. The doughs can be transformed to dense glassy solids of GO or graphene without long-range stacking order of the sheets, which exhibit isotropic and much enhanced mechanical properties due to hindered sliding between the sheets. GO dough is also found to be a good support material for electrocatalysts as it helps to form compliant interface to access the active particles.

## Abstract (web summary)

Graphene oxide (GO) dispersions may be used as starting materials for graphene-based architectures. Here, a malleable and versatile dough state of GO is discovered, completing the GO–water continuum, which can be diluted or converted to glassy GO or graphene solids without long-range stacking order with enhanced mechanical and electrochemical properties

## Introduction

Graphene oxide (GO), an oxidative exfoliation product of graphite, has gained significant interest as a building block to create graphene-based (a.k.a., reduced graphene oxide, r-GO) materials because of its excellent dispersibility in water and rich functionality^1–6^. GO dispersions are usually used as starting materials to construct various graphene-based architectures by solution-processing routes^3,7–12^. Direct fabrication of self-standing GO structures from dispersions or gels has been largely limited to thin films^3^ and lightweight foams^7,13^. A dough is a highly cohesive, malleable, and viscoelastic state of matter that can be readily reshaped without fracture, which is very useful for making free-standing three-dimensional structures^14,15^. As is with many other particulate materials, a semi-solid state of GO can be obtained by using additives with binder-like functions^16–18^, and additional processing steps are required if these additives need to be removed afterwards. It would be desirable to obtain ultrahigh concentration of GO (e.g., > 10 wt. %) in water to see if a dough state is accessible without the need for binders or cross-linkers, which has been challenging. For example, one could in principle obtain concentration of GO in water by drying a dilute solution, or by re-hydrating a dried GO solid. Evaporation can remove water from dilute GO solutions, but it is difficult to obtain very thick and uniform GO dispersions owing to GO’s tendency to go to the air-water surface, which hinders the evaporation process owing to their barrier properties^19–21^. On the other hand, adding small amounts of water to dried GO solids has also been difficult, because small aliquot of water tends to be absorbed locally, leading to non-uniform hydration. In addition, there had been some misunderstanding concerning the solubility of GO solids in water. It was once believed that GO papers, a very common form of GO solid made by filtration, are insoluble and cannot be re-dispersed in water^3,22^.

In an earlier work, we found that the insoluble GO papers obtained by filtrations are unintentionally cross-linked by the multivalent cationic contaminants released from some filter disks, and neat GO papers are indeed readily re-dispersed in water^23^ and can be glued together by water droplets^24^. It further suggests that the interlayer interactions between GO sheets can be weakened by water, and all neat GO structures should become dispersible in water. Based on this insight, here we report a “continuum” of GO–water mixtures, showing continuous transitions between four states, starting from a dilute dispersion, a thick gel, a malleable dough, and eventually to a dense solid as the water content decreases. This continuum finally completes the scope of GO–water mixtures with the long missing dough state, which typically has a mass fraction of GO ~10s of weight percent. GO doughs are found to be highly processable, can be shaped by common processing methods, and exhibit super extensibility^25^. This binder-free dough state of GO is a versatile material platform to fabricate bulk GO and graphene architectures with arbitrary shapes and tunable microstructures, including porosity and sheet alignment.

## Results

### GO–water continuum

**Fig. 1:**
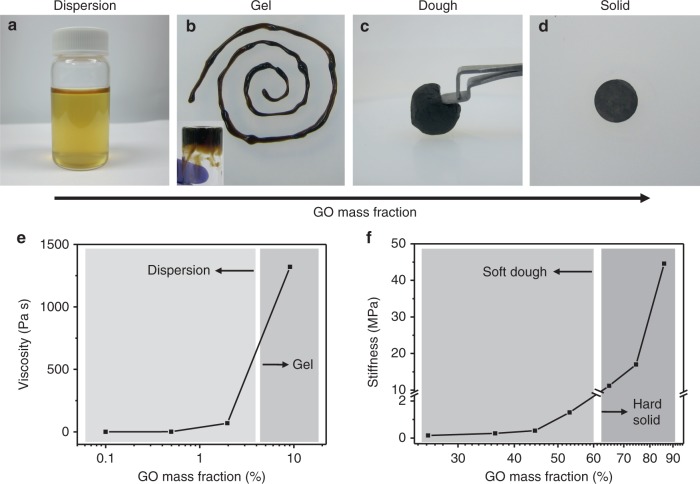
The GO–water continuum. Photos showing GO–water mixture transitioning from **a** a dilute dispersion, **b** a thick gel, **c** a malleable dough, to **d** a dense solid, as GO loading increases. **e** The transition from a dilute dispersion to a gel is characterized by a drastic increase in viscosity. **f** A dough-state GO is obtained when the mass fraction of GO exceeds 20 wt. %. GO dough is highly malleable until its mass fraction is increased to over 60 wt. %, after which the stiffness increases significantly

A system consisting of GO and water was prepared along a continuum of increasing concentration of GO, transitioning between four states starting with a dilute dispersion, a thick gel, a malleable dough, and a dense solid as shown in Fig. 1a–d. GO was synthesized by a modified Hummer’s method^26–28^. Powders of GO were obtained in the form of a filter cake after filtering a dispersion of purified sheets and drying in vacuum. Dilute dispersions (< 2 wt. %) were typically made by dispersing small pieces of dried GO filter cake in water. Direct preparation of higher-concentration dispersions from dried GO was difficult as the apparent volume of GO powders was already comparable or higher than the volume of water needed. Therefore, adding a small aliquot of water usually resulted in local, non-uniform hydration of GO powders. In order to obtain uniform GO–water mixtures with very high GO loadings, aerosolized water mists were applied to GO foams obtained by freeze-drying, which collapsed upon water uptake. This process allows uniform hydration of GO by a minute amount of water throughout the entire volume of the material, which has been difficult by other means. Further kneading and rolling of the collapsed foam turned the material into a dough state (Supplementary Figure 1). The dough state of GO can serve as a precursor to make high-concentration gels by dilution, or denser solids by drying.

Transitions between the four states were accompanied by significant changes in their rheological and mechanical properties. As the mass fraction of GO was increased, the dilute dispersion turned into a thick non-flowing gel as illustrated by the sample in an upside–down vial (Fig. 1b inset). Accordingly, a significant increase in viscosity was observed when the GO mass fraction exceeded 2 wt. % (Fig. 1e). Increasing the GO fraction to over 20 wt. % resulted in a viscoelastic dough state. Unlike the thick gels, the dough state can be kneaded and rolled on a surface without leaving extensive stains. This dough state can be easily deformed without fracture, and dried to form a dense GO solid that retained the shape (Fig. 1d). The dough state remained highly cohesive and processable until the mass fraction reached ~ 60 wt. %, after which the mixture became significantly stiffer, as characterized by a rapid increase of its compression modulus (Fig. 1f). As the loading of GO exceeded 60 wt. %, the solid became fragile and tended to fracture after compression.

Results of viscosity measurements of the GO–water mixtures including dispersions and gels are shown in Supplementary Figure 2, showing shear thinning behaviors that can be attributed to shear alignment of sheets and reduced tangling at higher shear rates^29^. Making the gel from the dough state is a lot more straightforward than other methods, and the rheological properties of the gels can be tuned over a large range, by simply adjusting the volume of water added. This allows customization of GO gels for a broad array of materials-processing techniques^30^, to create GO and graphene final products.

### Binder-free GO dough: a compact form for storage and transportation

**Fig. 2:**
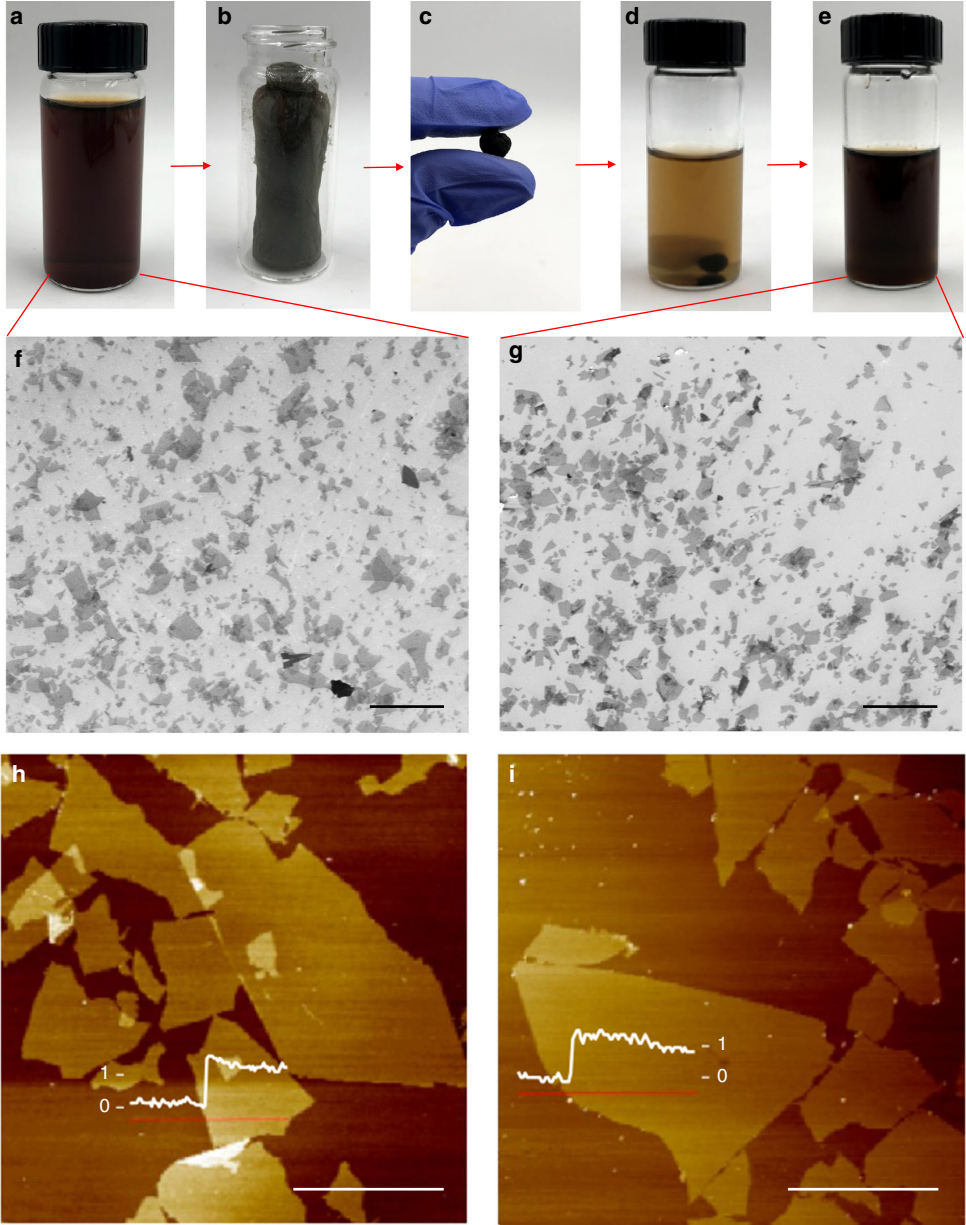
GO dough is readily re-dispersed in water. **a**–**e** Photos showing a GO sample converted from a dispersion to a foam, a dough, and back to the dispersion state. No significant changes in the sizes and morphology of GO sheets are observed before **f**, **h** and after **g**, **i** this process. **f**, **g** (scale bar = 20 µm) and **h**, **i** (scale bar = 10 µm) are images taken by SEM and AFM, respectively. Insets in **h** and **i** show height profiles of GO sheets in the unit of nanometers

The dough state is a highly versatile form of GO that is ideal for manufacturing. The high mass loading and compact form factors of GO doughs make them much more economical to store and transport. And as GO doughs are free of binders or cross-linkers, they can be readily re-dispersed in water to re-generate high-quality GO dispersion. The photos in Fig. 2a–e depict the transition of a GO sample through different states, starting from a dispersion, a foam to a dough state, which is then placed in water to yield dispersion again. As shown in the scanning electron microscopy (SEM) and atomic force microscopy (AFM) images (Fig. 2f, h), the as-made GO sheets have lateral dimension of ~ 5 to 20 µm and thickness of 1 nm. The dispersion was then freeze dried to yield a foam (Fig. 2b), which was subsequently hydrated and kneaded to obtain the dough state (Fig. 2c). The dough was then placed in water (Fig. 2d), gently stirred for ~ 10 mins, and sonicated in a tabletop sonicator for 2 mins, which results in a dispersion (Fig. 2e) with similar color, stability as the starting one (Fig. 2a). GO sheets spin coated from the final dispersion were found to have similar lateral dimension, thickness, and morphology (Fig. 2g, i) to these of starting GO sheets. These results suggest that converting GO dispersion into the much more compact dough state does not deteriorate the quality of GO sheets in terms of sizes and colloidal processability. Compared with other compact forms, such as dried powders or films, GO doughs are sufficiently hydrated to avoid spontaneous exothermal self-propagating reduction reaction^27^, and much safer to handle. GO doughs are also cleaner to handle than gels or pastes, owing to its high cohesivity and non-staining properties.

### Moldability and extensibility of GO dough

**Fig. 3:**
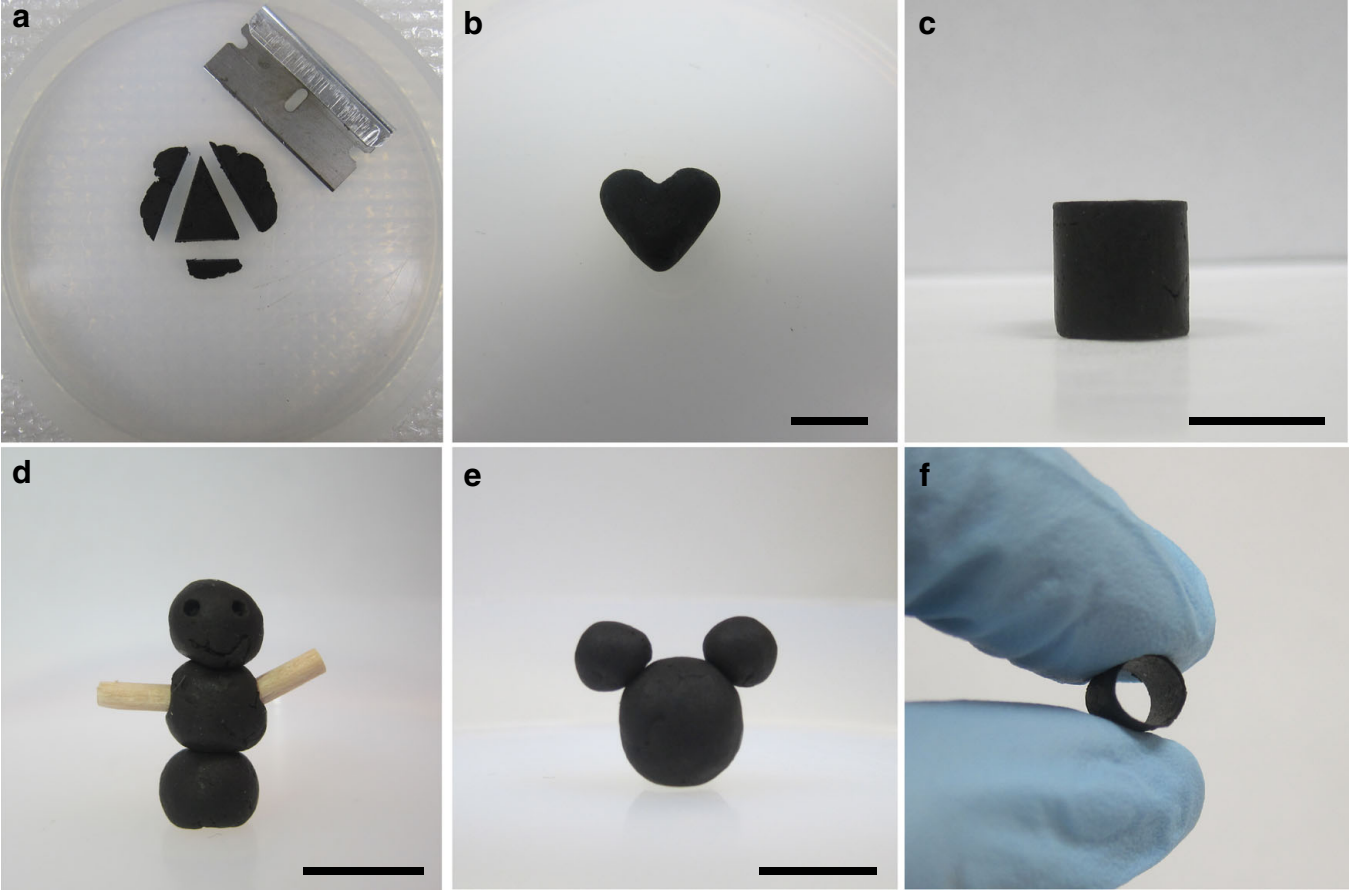
GO doughs are highly processable and versatile. GO doughs can be readily reshaped by **a** cutting, **b** pinching, **c** molding, and **d** carving. GO doughs can be easily connected together **d**, **e** or with other solid materials **d** using the wooden sticks as an example. **f** A tubular GO structure can be prepared by molding a GO dough around a rod, demonstrating the versatility of using GO doughs to make 3D architectures that are otherwise challenging to obtain. Scale bars in **b**, **c**, **d**, and **e** are 1 cm

**Fig. 4:**
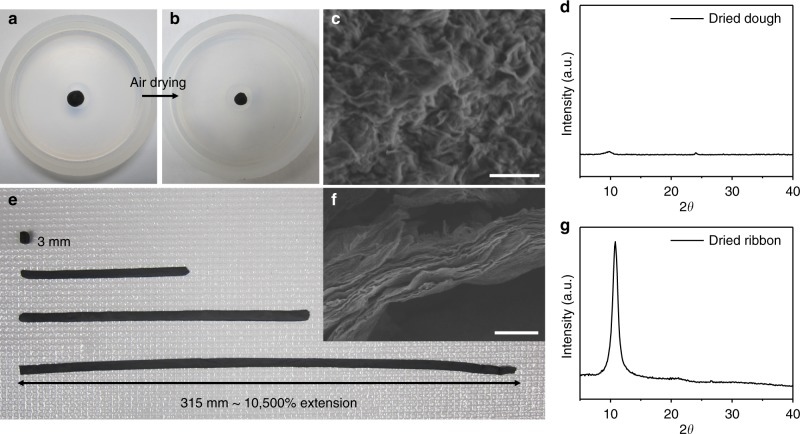
Super extensibility of GO doughs. Photos of a ball-shaped GO dough **a** before and **b** after drying in air, showing slight volume shrinkage. **c** Cross-sectional SEM image of the dried solid does not show lamellar microstructure, which is consistent with **d** the lack of a strong diffraction peak in the XRD pattern of the air-dried GO solid. **e** A short block of GO dough can be repeatedly cold rolled to a long GO ribbon with 10,500 % of extension. **f** Cross-sectional SEM image of the dried ribbon shows the development of lamellar microstructure, which is consistent with **g** the strong diffraction peak ~ 11° in the XRD pattern. Scale bar in **c** is 10 μm, and in **f** is 2 μm

The dough state of GO can be deformed into arbitrary form factors by common shaping methods, including cutting, shaping, molding, and carving (Fig. 3a–d). Pieces of GO doughs can be connected together simply by bringing them into contact followed by gentle compression (Fig. 3e). The versatility of GO dough allows unusual shapes to be made at ease, which has been hard by other means, as illustrated by the example of a tubular structure shown in Fig. 3f. As-made GO doughs isotropically shrinks upon air drying, yielding dense solids with disorderly packed sheets that are heavily wrinkled and crumpled (Fig. 4a–c). X-ray diffraction (XRD) pattern of the dried GO solid does not show an apparent peak ~ 11°, which is characteristic for lamellar GO structures with long-range stacking order of the sheets (Fig. 4d).

The GO dough can sustain extreme deformation without fracture, exhibiting super extensibility. A proof-of-concept is shown in Fig. 4e, where a 3-mm-long GO block was transformed into a long ribbon by cold rolling with lateral constraint. Two additional rolling steps were performed to further increase the length to 315 mm, corresponding to an elongation of 10,500 %. The extraordinary extensibility of the GO dough is attributed to both unfolding of the sheets and their sliding under shear. Indeed, the final cold-rolled ribbon has a lamellar microstructure (Fig. 4f). XRD pattern of the dried ribbon shows a strong diffraction peak, corresponding to an interlayer spacing of 8.18 Å, which is consistent with what has been reported for vacuum-filtered GO membranes^23^ (Fig. 4g). Preparing thick GO films by vacuum filtration is quite tedious due to the barrier properties of the sheets that makes filtration process self-limiting. GO dough could thus serve as a more versatile starting material for creating large-area GO films by rolling with the thickness controllable by the gap between the rollers.

The dough state of GO can be extended to a number of GO-based composites, leveraging GO’s surfactant properties^2,20^ to incorporate other components. For example, single-walled carbon nanotubes (SWCNTs) can be readily dispersed in water in the presence of GO, which can then be converted into a GO/SWCNT composite dough following the same procedure (Supplementary Figure 3). This GO/SWCNT composite dough is still highly cohesive and processable, which can also be cold rolled into a free-standing membrane, suggesting the potential of preparing composite doughs with various combinations of GO and functional materials based on the preparation method provided in this work.

### GO dough as a compliant support for electrocatalysts

The cohesivity, viscoelastic properties, and largely isotropic arrangement of sheets of GO dough makes it a suitable material platform to embed other particulate materials, such as fine particles for electrocatalytic purposes. Typically in electrocatalysis, catalytic particles need to be glued on electrodes with the help of binder materials (e.g., polymers or mineral oils) to improve adhesion, and conductive additives to improve charge transport (e.g., graphite and carbon black powders)^31–34^. The roles of these additives can be fulfilled by GO dough, which may simplify the electrode preparation. Here the dough can be readily deformed to yield geometrically compliant interface with the embedded materials, which after reduction to r-GO, provides robust electrical contact. The largely isotropic arrangement of GO sheets in the dough state also facilitates mass transport during catalytic reactions. The cohesivity of the dough also helps to preserve electrode integrity during reactions.

**Fig. 5:**
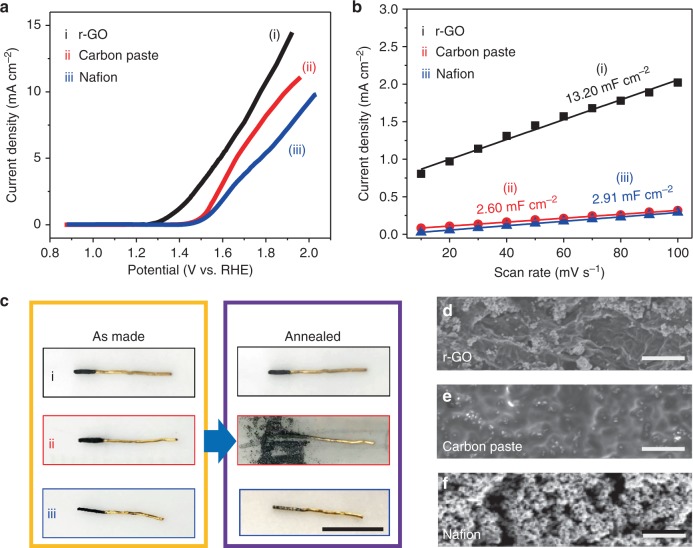
GO dough as a compliant support for electrocatalyst. **a** The linear sweep voltammetry curves of OER carried out in 0.1 m KOH using three different RuO_2_ electrodes made with annealed GO dough (i), carbon paste (ii), and Nafion (iii). **b** The electrochemical surface area of the three types of RuO_2_ electrodes. **c** Photos showing the three types of electrodes before and after annealing. The RuO_2_ particles are fixed on gold wires through the three types of binders. **d–f** The SEM images showing the surface morphology of the three types of electrodes. Scale bar in **c** is 1 cm, and in **d**, **e**, and **f** are 5 μm

A proof-of-concept experiment is carried out to compare the effectiveness of GO dough as a substrate for electrocatalytic oxygen evolution reactions (OER) with the conventional, widely used additive such as carbon paste or Nafion^35^. Commercial RuO_2_ particles are selected as the prototypical OER catalyst (see the details in Method), which was first embedded in a GO dough by direct mixing, followed by thermal annealing to convert GO to r-GO. As shown in Fig. 5a, the r-GO/RuO_2_ electrodes exhibit a small onset potential at 1.24 V, which is very close to the theoretical limit of OER at 1.23 V, suggesting highly efficient charge and mass transport enabled by the r-GO network to the active RuO_2_ particles. In contrast, the onset potentials for carbon paste/RuO_2_ and Nafion/RuO_2_ electrodes are 1.39 V and 1.41 V, respectively. The electrochemical surface area^36,37^ results (Fig. 5b) show that the highest value of the GO dough based electrodes (13.2 mF cm^−2^) is about four times higher than those delivered by electrodes using carbon paste (2.60 mF cm^−2^) and Nafion binders (2.91 mF cm^−2^), indicating much higher accessibility to the active RuO_2_ powders in the r-GO monolith support. The SEM images (Fig. 5d–f) of the three types of electrodes confirmed that RuO_2_ particles are indeed more uniformly dispersed in the r-GO support.

Using GO dough as catalyst support expands the types of electrode form factors that can be readily obtained. In conventional electrode composition, the active catalytic particles are mixed together with other particulate additives; therefore, they are typically deposited on a metal substrate by casting. In contrast, the r-GO/RuO_2_ electrode can be directly pressed onto a metal contact from their dough state. For example, the electrode shown in Fig. 5c (i) was made by direct wrapping of a cold-rolled slice of GO/RuO_2_ dough around a gold wire, followed by annealing. The resulting r-GO/RuO_2_ layer is quite robust, which not only sustained the annealing treatment, but also served as the efficient charge-transport contact for OER. In contrast, carbon paste and Nafion bound RuO_2_ electrodes tend to pulverize after annealing. Therefore, GO dough may be useful as a generic substrate for loading electrocatalyst particles for better evaluating their catalytic performance.

### Glassy GO solids

**Fig. 6:**
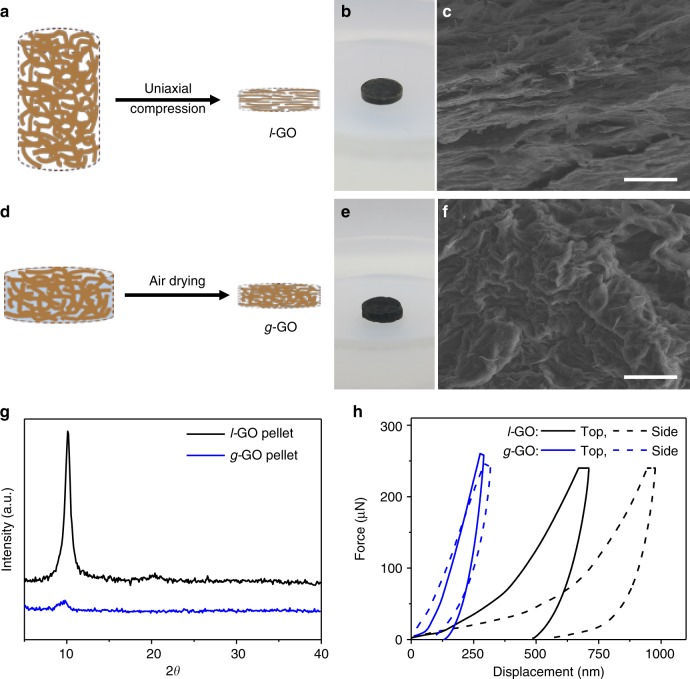
Glassy GO solid with enhanced and isotropic mechanical properties. **a–c** Uniaxial compression turns a freeze-dried GO foam to a GO pellet with lamellar microstructure, which is denoted as *l*-GO. **d**–**f** A glassy GO pellet made of disorderly packed sheets, denoted as *g*-GO, is made by first gently molding the dough into a pellet by hand, followed by drying in air. **g** XRD patterns and **h** nanoindentation curves of the two types of GO pellets. The *g*-GO pellet does not have a strong diffraction peak corresponding to lamellar ordering, and exhibits largely isotropic properties and higher hardness. Scale bars in **c** and **f** are 10 μm

The GO dough is a precursor to prepare dense GO solid with unique isotropic microstructure and properties. Typically, GO structures exhibit lamellar microstructures with long-range stacking order of GO sheets^38^, which leads to orientation dependent properties. However, as the dough is obtained by a small degree of hydration of GO foam, followed by kneading, the sheets are not aligned. During drying, the dough experiences isotropic capillary compression and gradually densifies by squeezing and deforming the GO sheets. The final microstructure is made of densely packed, heavily deformed sheets without apparent long-range stacking order, which bears strong resemblance to that of pistachio shells^39^. To study the effect of sheet alignment in the properties of dense GO solids, two bulk pellets with similar size, shape, and density but with different types of microstructures were prepared. One pellet was made by compressing a freeze-dried GO foam at 200 MPa to induce alignment of the GO sheets (Fig. 6a, b). Such uniaxial compression indeed rendered the resulting pellet a lamellar microstructure (Fig. 6c) with a strong XRD peak corresponding to an interlayer spacing of 8.74 Å (Fig. 6g). The other pellet was made by gently molding a GO dough into the same shape, followed by drying in air (Fig. 6d, e). Cross-sectional SEM image of the dried pellet did not show any obvious lamellar ordering (Fig. 6f), which is consistent with the lack of a strong diffraction peak ~ 11° in the XRD pattern (Fig. 6g). Both types of pellets have similar densities measured to be ~ 1.5–1.6 g cm^−3^. The pellet with lamellar ordering is denoted as *l*-GO pellet, whereas the one with disordered sheets is denoted as glassy GO (*g*-GO) pellet.

Indentation tests were applied to study the mechanical properties of the two types of pellets at both their top and side surfaces. The corresponding force–displacement curves are shown in Fig. 6h. In the *l*-GO pellet, the sheets are aligned in parallel to the top surface. Indentation on the side should encounter smaller resistance, because the load can easily cause sliding, deformation, or even partial opening of the lamellar structure. Indentation along the top surface should encounter higher resistance, as the force is normal to the sliding direction of sheets. Indeed, the *l*-GO pellet is significantly softer on the side with a measured hardness of 13.38 ± 3.02 MPa, in comparison with 25.29 ± 5.20 MPa measured on the top surface. Contrasting the anisotropic mechanical property of the *l*-GO pellet, the hardness of *g*-GO pellet does not exhibit significant orientation dependence, with a hardness of 133.37 ± 14.76 MPa measured on the top surface and 117.15 ± 12.66 MPa on the side. Similar displacement values were obtained when indenting the *g*-GO pellet from both its top surface and side. The hardness of *g*-GO pellet is drastically higher than those of *l*-GO. This is because the *g*-GO pellet is made of entangled and heavily crumpled sheets, which hinders sliding, making it much more resistant to deformation. Taken together, these results show that GO solids of similar size, shape, and density can exhibit significantly different properties owing to different sheet alignment. The GO dough is thus very suitable for creating bulk GO materials with isotropic properties.

### Graphenic glass

**Fig. 7:**
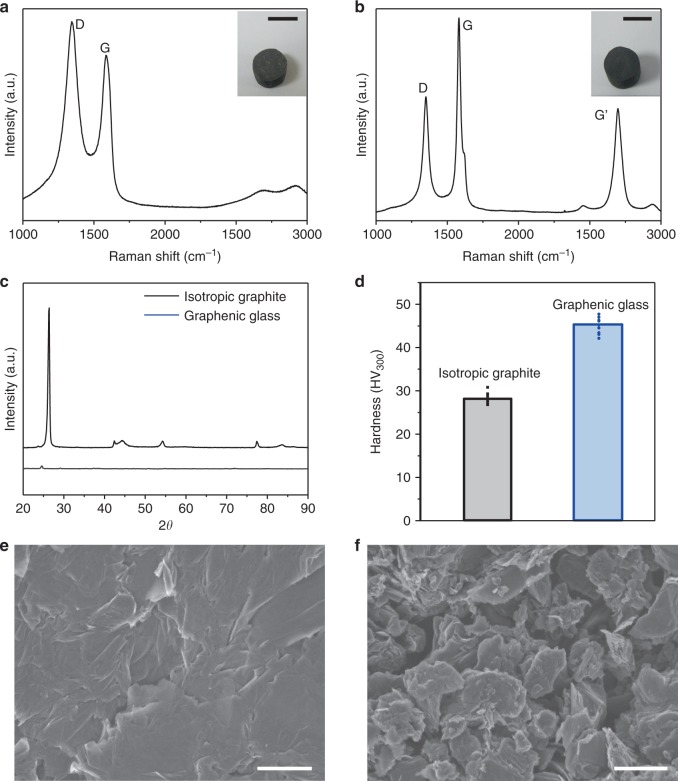
Glassy graphene solid. Raman spectra of **a** a graphenic glass and **b** an isotropic graphite, showing that the graphenic glass is composed of more defective graphene sheets (insets of **a**, **b** are photos of the two cylindrical samples). **c** XRD patterns show that the isotropic graphite is made of highly crystalline graphite grains while the graphenic glass, at similar density, does not have obvious long-range stacking order of the sheets. **d** Vickers hardness tests show that the graphenic glass is harder than the isotropic graphite, which can be attributed to **e** its smoother microstructure with finely distributed free volume (i.e., voids). In contrast, **f** the isotropic graphite has a much rougher and more granular microstructure, with much larger voids. Scale bars in a and b insets are 5 mm, and in **e** and **f** are 1 μm

As the dough state makes isotropic glassy GO solids readily accessible, it should also lead to glassy graphene solids after reducing GO. In such solids, the graphene sheets are densely but disorderly packed, exhibiting weak long-range stacking order. Therefore, they are named graphenic glass, taking inspirations from bulk metallic glass^40^. To prepare such glassy graphene solid, a GO dough (c.a., 50 wt. %) was first molded into a cylindrical shape, and then reduced by HI vapor^41^, followed by washing with ethanol. The dried solid was further hot pressed under 50 MPa at 800 °C to a final density of 1.71 g cm^−3^ (Fig. 7a, inset), which is comparable to the densities of compressed graphite materials^42^. XRD pattern of the resulting graphenic solid only shows a very weak diffraction peak ~ 26°, indicating the stacking of graphene sheets is rather disordered (Fig. 7c).

As is with glassy GO, sheet sliding is also significantly hindered in graphenic glass, which should make them harder than their lamellar counterpart. For comparison, a commercially available high strength graphite rod with a similar density of 1.78 g cm^−3^ (Fig. 7b, inset) was selected as a control sample. Such bulk graphite materials are typically made by isostatically densifying fine graphite powders with a binder, followed by calcination at elevated temperatures (e.g., 2500–3000 °C), and have widespread applications in areas including semiconductor industry and nuclear reactors^43^. Although the graphite grains still have anisotropic properties, the bulk solid is considered isotropic owing to the averaging effect of randomly oriented graphite grains. The isotropic graphite cylinder sample used in this work has relatively fine grains ~ 5 µm, and is known for its comparatively high strength and hardness, which makes them suitable for making die sets for hot-pressing applications. In contrast, the glassy graphene solid is made of much weaker and less graphitized r-GO sheets. Indeed, Raman spectra (Fig. 7a, b) show that the r-GO sheets in the glassy graphene solid is a lot more defective (with an I_D_/I_G_ ratio of 1.21) than those in the bulk graphite sample (with an I_D_/I_G_ ratio of 0.61) (Fig. 7a, b). Since the two samples have similar densities, and the isotropic graphite is much more graphitized and made of harder grains, one would expect the isotropic graphite should be harder than the graphenic glass. However, Vickers hardness tests show that the graphenic glass (with a hardness of 45.14 ± 1.84 HV_300_) is actually harder than the isotropic graphite (with a hardness of 28.01 ± 1.29 HV_300_) (Fig. 7d). SEM images taken on their polished surfaces reveal drastically different microstructures. The graphenic glass has a relatively smooth texture, while the isotropic graphite shows a much rougher and more granular microstructure with visible voids (Fig. 7e, f). As the two samples have similar densities, they should have comparable level of free volume. Therefore, the smoother microstructure of the glassy graphene suggests that its free volume must be more finely divided and more uniformly distributed throughout the material. The higher hardness of the glassy graphene can be attributed to the finely distributed free volume and entangled network of the sheets within the solid, which renders it higher resistance to plastic deformation. In contrast, although the isotropic graphite has harder grains, it also has more segregated free volumes (i.e., voids), which cannot hinder sliding of the graphite grains, leading to lower hardness. The hardness of the graphenic glass is also higher than otherwise densified graphene materials including graphene-CNT hybrid solid made by pressure assisted welding^44^, and is comparable to that of the bulk graphene nanoplatelets fabricated by spark plasma sintering^45^. Dense graphene monoliths can be obtained from hydrogels made from hydrothermal reactions, and their size and shape are limited by the volume and shape of the hydrothermal reactors^46–48^. Therefore, the GO dough is a more versatile precursor to make bulk graphitic materials with arbitrary form factors, which does not rely on the use of binders, extensive mechanical compression, or high-temperature treatment.

## Discussion

The GO–water continuum is now completed with a long missing dough state, which can be readily converted to thick gels by dilution or dense solids after drying. The dough state of GO is obtained without any binder and is highly processable. It can be deformed into arbitrary shapes and retains the shapes after drying. GO dough can act as highly compliant support for electrocatalytic particles, and after reduction to r-GO, offers high quality electrical interfaces to access the active catalyst during electrocatalysis. The dough can yield dense GO solids with disorderly packed sheets as a result of isotropic capillary compression during drying. Sheets in such glassy GO solids are crumpled and interlocked, leading to isotropic properties and higher hardness.

Glassy graphene solids can be made similarly after reducing GO sheets, which also exhibit isotropic properties. The graphenic glass, although made of less graphitized r-GO sheets, is even harder than commercial high-strength graphite materials owing to its unique microstructure limiting sheet sliding. This proof-of-concept suggests the promise of the GO dough for fabricating high performance graphenic glass materials in the widespread applications of current bulk graphite materials.

The dough state of GO also brings some benefit to its storage and transportation during manufacturing owing to its high mass loading and compact form factors. GO doughs can also turn into high-quality dispersions simply by adding water. It is a safer form than dried powders or films^27^ owing to its higher degree of hydration and cohesivity, and a more convenient form to handle than gels and paste due to its non-staining properties. GO doughs can also be easily converted to dense GO or r-GO foams with tunable density and porosity. Their extraordinary processability can potentially make GO and graphene as easy to use as common engineering materials to create desirable size, shape, and structures in bulk forms^49^.

## Methods

### Preparation of GO dispersions

GO was synthesized using a modified Hummers method with a two-step purification process^26–28^. In a typical reaction, 6 g of graphite (Asbury, #2139), 5 g of K_2_S_2_O_8_, 5 g of P_2_O_5_, and 25 mL of H_2_SO_4_ were stirred together at 80 ± 5 °C. Next, the dispersion was diluted and filtered with filter papers (Whatman, Grade No. 3). The pre-treated graphite powders were then collected and dispersed in 230 mL of H_2_SO_4_. After that, 30 g of KMnO_4_ was slowly added. The mixture was kept at 35 ± 5 °C for 2 h and then slowly diluted with 0.5 L of deionised water, followed by the addition of 30 mL of 30 % H_2_O_2_. In the purification process, the mixture was first filtered using polytetrafluoroethylene membrane (Millipore) and rinsed with 3.4 % HCl solution. After drying in vacuum, the as-prepared GO was re-dispersed and washed in acetone, and eventually filtered and dried again to yield GO cakes. The as-received GO cakes can be readily dispersed in water by shaking, which can be accelerated by gentle sonication (e.g., a few minutes in a tabletop sonicator). Solid chemicals were purchased from Sigma-Aldrich, and liquid chemicals were purchased from Fisher Chemical. All chemicals were used as received.

### Preparation of freeze-dried GO foams

In a typical freeze-drying procedure, 10 mL of GO dispersions at a concentration of 5 mg/mL were prepared in glass vials. Such GO dispersions were then immersed in a liquid nitrogen bath to freeze for 10 min. After GO dispersions were frozen, the vials were then transferred to a freezedryer, and porous and spongy GO foams were formed after the ice in frozen GO dispersions was completely sublimated (typically in 1–2 days).

### Preparation of GO doughs

GO doughs were obtained by partially hydrating freeze-dried GO foams using water mist, which can be achieved by a handheld aerosol generator or blowing air through a hot water bath to carry moisture (Supplementary Figure 4). The color of the foam darkens upon hydration, which can be used to monitor degree of hydration. Kneading and rolling the hydrated GO foams yielded GO doughs with GO mass fractions generally between 40–60 wt. %. The degree of hydration can be further tuned by adjusting the amount of additional water added in the GO doughs.

### From GO doughs to gels

Further hydrating the GO dough turned it into a sticky and gel-like material, which can be directly extruded via a syringe. The viscosity of the gel can be tuned at ease by adjusting the amount of water added into the dough.

### Preparation of r-GO/RuO_2_ electrode

RuO_2_ particles (Alfa Aesar) were blended in a GO dough (50 wt. % GO content) using a mixer (Thinky AR-100) with a RuO_2_: GO weight ratio of 3: 7. The resultant dough was cold rolled into a sheet and then wrapped around an Au wire (0.5 mm in diameter). The diameter of the final electrode was ~ 1 mm. The GO/RuO_2_ electrode was then freeze dried followed by thermal annealing at 250 °C for 2 h (heating rate: 1.5 °C min^−1^) under argon to reduce GO. The RuO_2_: r-GO weight ratio in the final electrodes is ~ 3: 3.5.

### Preparation of carbon paste/RuO_2_ electrode

Based on previously reported procedures^31–33,50,51^, carbon paste was prepared by mixing graphite powders (150 mesh) and mineral oil with a weight ratio of 70:30. Next RuO_2_ was added to the paste with a weight ratio of 3:3.5. The carbon/RuO_2_ paste was then coated on an Au wire of 0.5 mm in diameter. Stability of the paste on the electrode was tested by annealing at 310 °C under Ar.

### Preparation of Nafion/RuO_2_ electrodes

Nafion was purchased from Sigma-Aldrich (5 wt. % in water). The Nafion/RuO_2_ electrode was prepared by mixing 4 mg RuO_2_ (i.e., identical to that used in the GO dough based electrodes) with 2 μL of Nafion solution (0.5 wt. % diluted by ethanol). The mixture was then coated on an Au wire (0.5 mm in diameter), and dried at room temperature overnight. Stability of the Nafion/RuO_2_ coating on Au was tested by annealing at 200 °C under Ar.

### Electrochemical measurements

The electrochemical tests were conducted using a three-electrode cell connected to an Autolab PGSTAT302N station. RuO_2_-coated Au wires were used as the working electrodes, a Pt plate was used as the counter electrode, and Ag/AgCl as the reference electrode. Linear sweep voltammetry was conducted at a scan rate of 5 mV s^−1^ in 0.1 M KOH. The potentials were transferred to reversible hydrogen electrode (RHE) via following equation:

$${\mathrm{E}}_{{\mathrm{RHE}}}{\mathrm{ = E}}_{{\mathrm{Ag/AgCl}}} + 0{\mathrm{.1976 + 0}}{\mathrm{.059 \times pH}}$$

Electrochemical surface areas were measured using cyclic voltammetry from 1.025 to 1.125 V (vs. RHE) in 0.1 M KOH. The scan rates were carried from 10 to 100 mA s^−1^ with an interval of 10 mV s^−1^. The slopes of current density (at 1.12 V) as the function of scan rates were taken to be the double layer capacitance (C_dl_), which was multiplied by 1000 to calculate the electrochemical surface areas (mF cm^−2^).

### Glassy GO solids

GO doughs were molded into arbitrary shapes such as a heart, a ball, and a pellet. Freeze-drying preserved their size and shape, leading to high-density foams. Air drying or vacuum drying the GO doughs resulted in dense GO solids with isotropically packed sheets that also became crumpled. Such solids are named glassy GO solids due to lack of long-range stacking order reflected in their XRD patterns.

### Preparation of lamellar and glassy GO pellets

High-density lamellar GO pellets were prepared by directly compressing freeze-dried GO foams in a stainless steel die at a pressure of 200 MPa. Glassy GO pellets were prepared from GO doughs, which were first molded into pellets by gentle pressing in a Teflon die by hand, followed by drying in air. The two pellets were fractured in order to study their mechanical properties perpendicular to the top surface of the pellets.

### Graphenic glass

GO doughs with mass loading over 50 wt. % were reduced using hydroiodic acid vapor^41^. GO doughs were put in a glass petri dish, and the petri dish was placed in a sealed beaker containing 1 mL of HI solution, which was then heated to ~ 60 °C for 2 days. The reduction process yielded r-GO solids with volume shrinkage. The r-GO solids were then washed with ethanol, after which they were loaded in a graphite die and hot pressed at 800 °C under a pressure of 50 MPa for 10 min. The heating rate was 5 °C min^−1^.

### Isotropic graphite

Based on a survey of disclosed isotropic graphite properties, two isotropic graphite rods with higher strengths and hardness were purchased from MERSEN USA Greenville-MI Corp. and McMaster-Carr for hardness measurement. The sample from MERSEN USA Greenville-MI Corp. was measured to have a higher hardness, and was chosen as a control sample.

### Characterization

Elemental analysis of the as-made GO was done by combustion elemental analysis (Atlantic Microlab, Norcross, GA), and the weight percentage of C, H, Cl, and S were found to be 42.58 %, 2.50 %, 0.84 %, and 3.68%, respectively. SEM images were obtained by field emission scanning electron microscopy (NOVA NanoSEM 600 and Hitachi SU8300). AFM images were acquired with a Park Systems XE-100 AFM under tapping mode. The XRD patterns were collected with a Rigaku Dmax powder diffractometer with Cu Kα radiation (*λ* = 1.5418 Å) at 40 kV. Static mechanical uniaxial compressive tests were conducted with a dynamic mechanical analyzer (EltroForce 5500, BOSE). Rheological characterization was performed on a rheometer (Anton Paar, MCR 302) using a stainless steel cone-and-plate geometry at room temperature. Hardness was obtained via nanoindentation (Hysitron TI 950 Triboindenter) using a standard three-sided pyramidal Berkovich probe with contact areas of ~ 1–20 µm^2^. As the grains of commercial isotropic graphite are bigger than the nanoindentation tip, its hardness was measured using Vickers hardness tests on a Duramin 5 (Struers) microhardness tester equipped with a square-based pyramidal diamond indenter. An indentation load of 300 gf and a holding time of 10 s were applied with contact areas of ~ 6000–12000 µm^2^. Raman spectroscopy was performed with a laser beam wavelength of 532 nm (Acton TriVista CRS System).

## Supplementary information

Supplementary information

## Data Availability

The data that support the findings of this study are available from the authors upon reasonable requests.
