# Supplementary material for: Binder-free graphene oxide doughs
Source: Nat Commun. 2019 Jan 24;10:422. doi: 10.1038/s41467-019-08389-6 (PMC6345773; doi:10.1038/s41467-019-08389-6)
Supplement: Supplementary file 1 — Supplementary information [file 41467_2019_8389_MOESM1_ESM.pdf]

Supplementary Information

Binder-free Graphene Oxide Doughs

Che-Ning Yeh,[1] Haiyue Huang,[1] Alane Tarianna O. Lim,[1] Ren-Huai Jhang,[1,2] Chun-Hu Chen[1,2] and Jiaxing Huang[1]**

1. Department of Materials Science and Engineering, Northwestern University, Evanston, IL 60208, USA

2. Department of Chemistry, National Sun Yat-sen University, Kaohsiung, Taiwan, 80424

*Email: (J.H.) jiaxing-huang@northwestern.edu and (C.C.) chunhu.chen@mail.nsysu.edu.tw

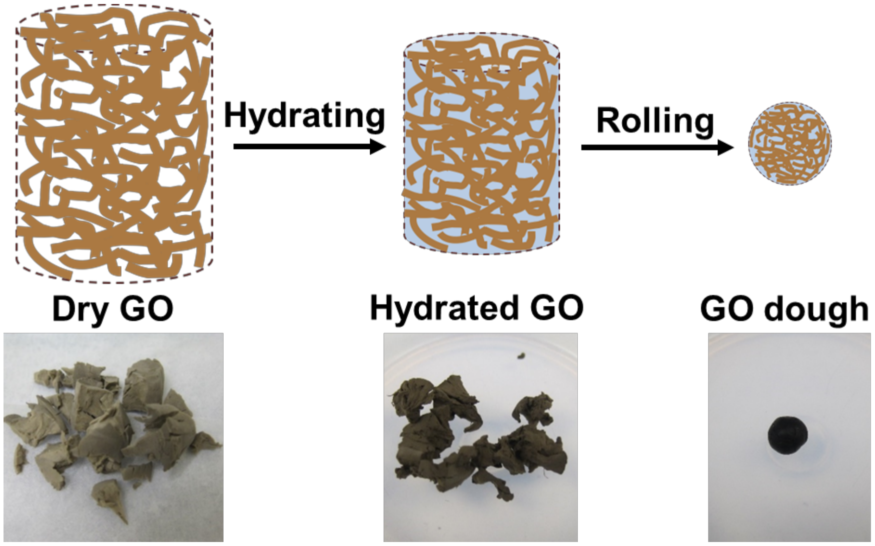

Supplementary Figure 1. Preparation of GO doughs. Schematic diagrams and photos showing that GO doughs were obtained by hydrating dry GO foams with aerosolized water mist. After hydration, GO foams collapsed and turned into hydrated GO, which can then be kneaded and rolled into a GO dough.

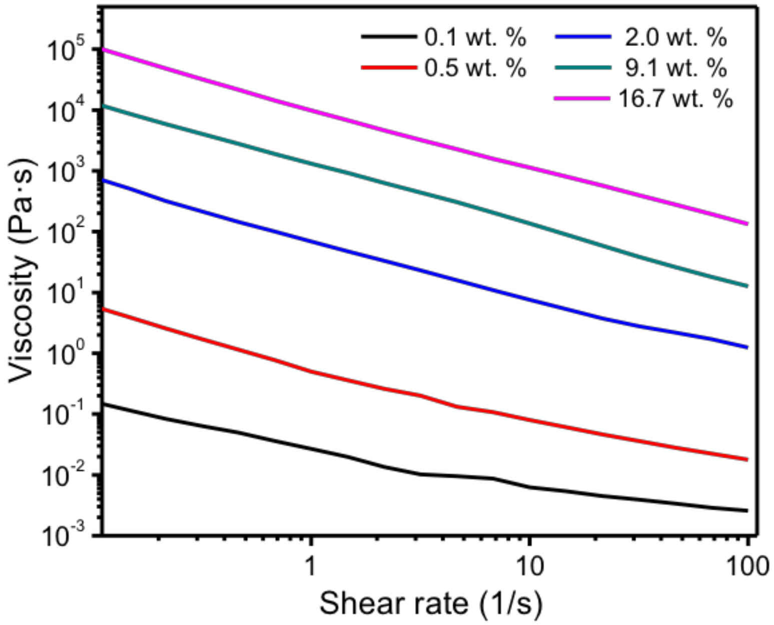

Supplementary Figure 2. Rheological properties of the GO-water dispersions and gels. The evolution of viscosity against shear rate for the GO-water at various GO mass fractions, showing that they are shear thinning and that their viscosities can be tuned over a wide range of values.

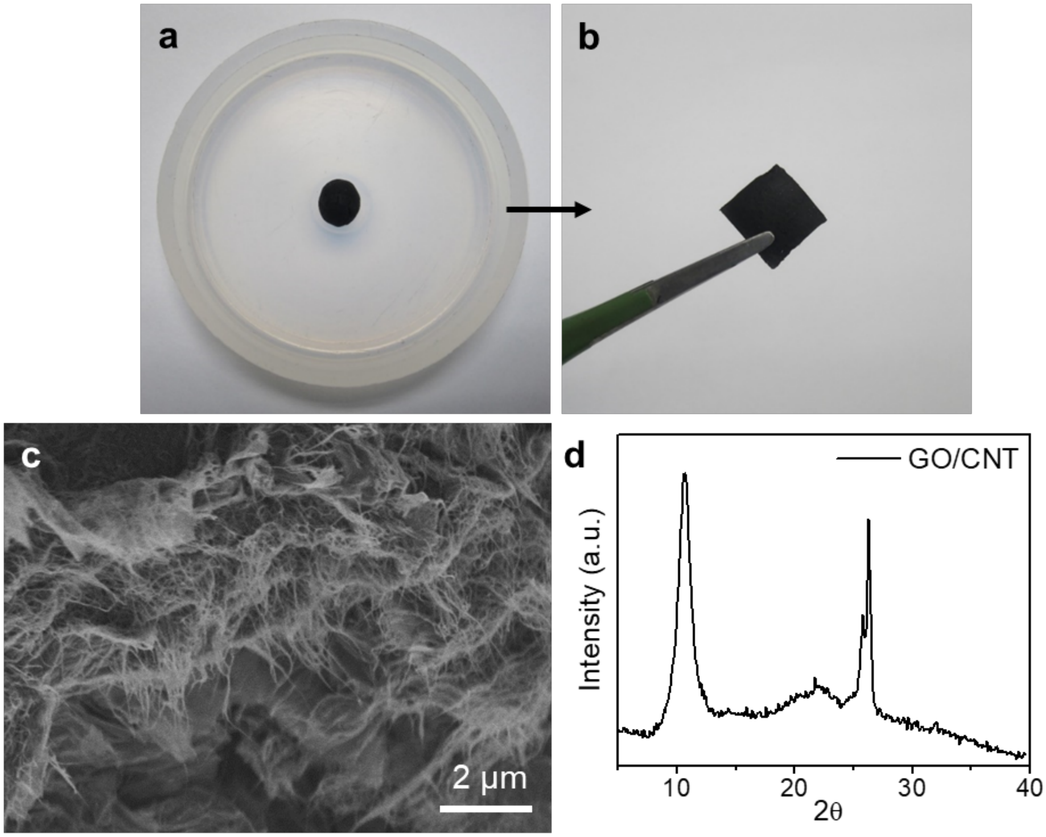

Supplementary Figure 3. Preparation of GO/SWCNT composite dough and membrane. GO dough can serve as a versatile material platform to incorporate other components. For example, single-walled carbon nanotubes (SWCNTs) were added to an aqueous GO dispersion to form a uniform GO/SWCNT dispersion, which can then be converted into (a) a GO/SWCNT composite dough. Such dough was also highly processable, and can be cold rolled into (b) a free-standing membrane. (c) Cross-sectional SEM image of the dried composite shows SWCNTs sandwiched between GO layers and (d) the XRD pattern shows characteristic peaks of GO (around 11º) and SWCNT (around 26º).

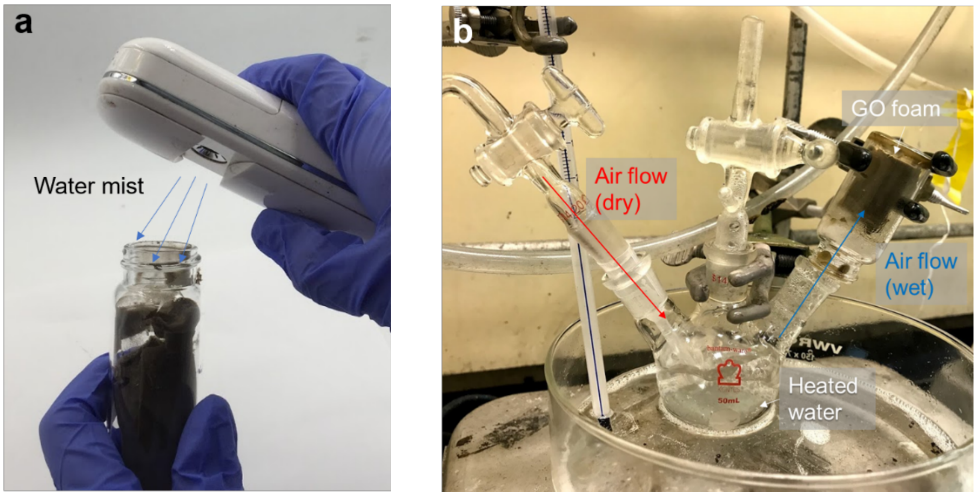

Supplementary Figure 4. GO foam can be hydrated using a number of mist/vapor delivery methods, such as by (a) spraying with an aerosol mist generator or (b) blowing hot water vapor into the foam.
